# Development of an orthotopic model of human metastatic prostate cancer in the NOD-SCIDγ mouse (*Mus musculus*) anterior prostate

**DOI:** 10.3892/ol.2015.3522

**Published:** 2015-07-22

**Authors:** FEDERICO F. CIFUENTES, RODRIGO H. VALENZUELA, HÉCTOR R. CONTRERAS, ENRIQUE A. CASTELLÓN

**Affiliations:** 1 Laboratory of Molecular and Cellular Andrology, Physiology and Biophysics Program, Institute of Biomedical Sciences, Faculty of Medicine, University of Chile, Santiago 8380453, Chile; 2 Department of Animal Pathology, Faculty of Veterinary and Animal Sciences, University of Chile, Santiago 8820808, Chile

**Keywords:** prostate cancer, NOD-SCIDγ mouse, anterior prostate, primary tumor, metastasis

## Abstract

Prostate cancer is one of the most prevalent oncological diseases in males worldwide, and the mortalities resulting from this type of cancer are mainly due to metastasis. The most common models for the study of metastasis are transgenic and immunocompromised mice, which enable the study of the metastatic process in a controlled way by the injection of prostate cancer cells into the mice. In the present study, NOD-SCIDγ mice were injected orthotopically with PC3 cells in the anterior prostate in order to establish a metastatic model. The results demonstrated the development and growth of a primary tumor that preceded the formation of micrometastases in the lung, liver and pancreas, followed by macrometastases in the liver. This model adequately represents the dynamics of the metastatic process, and may be useful for novel therapeutic assays and post-surgical relapse studies.

## Introduction

*In vivo* models of cancer enable the study of tumor development and the dissemination of cancer cells in a complex organism, thereby simulating the process observed in human (1). These models are useful in a preclinical setting, as they facilitate the investigation of the biology of cancer and its response to therapeutic agents (2,3). The development of mouse models of metastatic prostate cancer (PCa) is required for the study of the dissemination patterns of tumor cells, and to explore novel therapeutic approaches (4).

The mouse (*Mus musculus*) is the preferred animal for *in vivo* models of cancer, due to its homogeneous biological features and the short time over which cancer develops in this organism (5). Thus, numerous mouse models of PCa have been developed, and these can be grouped into the 2 following categories: Transgenic or knockout (6); and immunocompromised models (7). The nude mouse, which lacks thymic development and presents functional and quantitative defects in T lymphocytes, is a widely used immunocompromised model (7). The severe combined immunodeficiency (SCID) mouse presents non-functional T and B lymphocytes and preserved innate immunity (7,8). In 1995, the SCID mouse was combined with the non-obese diabetic (NOD) mouse, and the result was a mouse with no T or B lymphocytes, defects in natural killer cell function, absence of circulating complement and defects in the differentiation and function of antigen-presenting cells (9). The NOD-SCIDγ mouse was developed later, which is a variant with a lower tendency for lymphocyte leakage than the NOD-SCID mouse (10).

Several PCa cell lines, including PC3 (7), DU145 (7) and LNCaP (11), and tissue fragments from human primary tumors (8) have been used to study PCa in the NOD-SCID mouse. The injection sites in this model include subcutaneous, renal subcapsular, bone marrow and orthotopic injection (7,11). Among these, the most effective procedure for producing metastases has been the orthotopic route. Metastases may develop in the lung, liver, lymph nodes, kidney and pancreas (7,11). The orthotopic model is associated with the release of viable circulating tumor cells (12).

The mouse prostate is composed of 4 lobes, including the anterior (also known as the coagulating gland), ventral, dorsal and lateral lobes. The latter 2 are jointly referred to as the dorsolateral lobe (13). This is the most common site for the orthotopic implantation of cells. The anterior lobe is a paired elongated structure, with each branch running along the medial aspect of the seminal vesicle of each side. Histologically, this lobe is formed from several dilated acinar structures lined by 2 layers of low cuboidal epithelium. The interstitium is abundant and consists of a loose matrix with scattered stromal cells and blood vessels. The anterior lobe shares molecular and histological characteristics with the other prostate lobes, particularly with the dorsolateral lobe, which in turn is regarded as the most similar lobe to the peripheral zone of the human prostate, where cancer usually arises (14,15).

With regards to research into PCa, there is a limited number of studies that have investigated the anterior prostate as a site for the injection of cells (16). In the present study, a modification of the traditional method of orthotopic implantation of cancer cells was developed using the anterior prostate as the site of injection. The expression signature in this lobe is similar to that observed in the dorsolateral lobe, and is amenable to surgical resection (14). This approach may present advantages for future studies on post-surgical local relapse. The aim of the current study was to establish a primary tumor from human PCa cells and to follow its growth and the development of distant metastases by anatomopathological and molecular techniques.

## Materials and methods

### 

#### Prostate cancer cell line and culture conditions

The PC3 cell line (CRL-1435; American Type Culture Collection, Manassas, VA, USA), a human prostate carcinoma cell line established from a bone metastasis, was used in the present study. When cultured, these cells present adherent growth and epithelial morphology. This line is negative for androgen receptor and posesses a high tumorigenic capacity. The PC3 cells were cultured in DMEM/F12 (Life Technologies, Grand Island, NY, USA) supplemented with 10% fetal calf serum (Life Technologies), and maintained at 37°C in a humidified incubator with 5% CO_2_ atmosphere.

#### Animals

NOD-SCIDγ mice (NOD.Cg-Prkdcscid Il2rgtm1Wjl/SzJ; Jackson Laboratory, Sacramento, CA, USA) were obtained from the high safety animal facility, Faculty of Medicine, University of Chile (Santiago, Chile), and maintained in a laminar flow room under specific pathogen-free conditions. All the food, water and litter were sterilized prior to use. The temperature and humidity were controlled at 20–21°C and 50–60%, respectively. Daily light cycles consisted of 12 h light and dark. The cages were fully cleaned once or twice per week. The animals were manipulated under sterile conditions.

#### Experimental design

A total of 7 groups of 2 mice each were injected with 50 µl phosphate-buffered saline (PBS) or 10^6^ PC3 cells suspended in PBS (total volume of 50 µl), as indicated in Table I.

#### Orthotopic and subcutaneous implantation

The animals were anesthetized with an intraperitoneal injection of 10 mg/kg xylazine (Virbac, Santiago, Chile) combined with 100 mg/kg ketamine (Merial Sanofi, Santiago, Chile) in a total volume of 200 ml of sterile water. The implantation was performed under surgically sterile conditions. For the orthotopic injection, the abdomen was cleaned with iodine solution (DifemPharma, Santiago, Chile), and a 1-cm midline incision was created to expose the prostate gland. A 30-gauge needle and a 1-ml disposable syringe were used for the injection of the cell suspension. The needle was inserted into the middle portion of the right anterior lobe, and then was directed towards the cranial portion of the lobe, to reduce the risk of accidental implantation in the opposite lobe. The abdominal wound was closed in 2 layers with 6/0 absorbable surgical suture.

Subcutaneous implantation is an easy and secure way for injecting tumor cells. However, it may not be representative of prostate cancer and may not result in metastatic disease (7). In order to compare the tumor growth and metastatic capacity of a subcutaneous tumor, 50 µl cell suspension (10^6^ PC3 cells in PBS) were injected into the subcutaneous tissue of the right lateral abdomen of the mice without anesthesia, using a 30-gauge needle and a 1-ml disposable syringe.

At the end of the experiment, the animals were euthanized in a CO_2_ chamber. A post-mortem examination was performed to observe macroscopic changes, presence and localization of tumors and to obtain tissues for the subsequent microscopic and molecular analyses.

#### Histology and immunohistochemistry

The tissues were fixed by submersion in neutral-buffered formalin (Sigma-Aldrich, Schnelldorf, Germany) for 24 h, and then trimmed and placed in histological cassettes for dehydration, inclusion in paraffin (Sigma-Aldrich) and staining with hematoxylin and eosin (Merck KGaA, Darmstadt, Germany). The tissues were subjected to an indirect immunoperoxidase method as follows: Antigen retrieval was achieved by exposing the samples to 90°C for 30 min in citrate buffer (10 mM, pH 6.0; Sigma-Aldrich), followed by overnight incubation at 4°C with the mouse monoclonal anti-human mitochondria antibody (ab3298; Abcam, Cambridge, MA, USA). Next, the samples were treated using a streptavidin-biotin detection method (using a Histostain-Plus Bulk Kit, a LAB-SA Detection System and a DAB-Plus Reagent Set; Invitrogen Life Technologies, Camarillo, CA, USA) followed by hematoxylin counterstaining. The images of the tissues were obtained with a DM3000 laboratory microscope (Leica Microsystems, Inc., Buffalo Grove, IL, USA).

#### Bioethical approval

All the procedures involving animals were approved by the Bioethical Committee for Animal Research of the Faculty of Medicine, University of Chile (protocol CBA#0487 FMUCH).

## Results

### 

#### Necropsy

**Figure 1.**
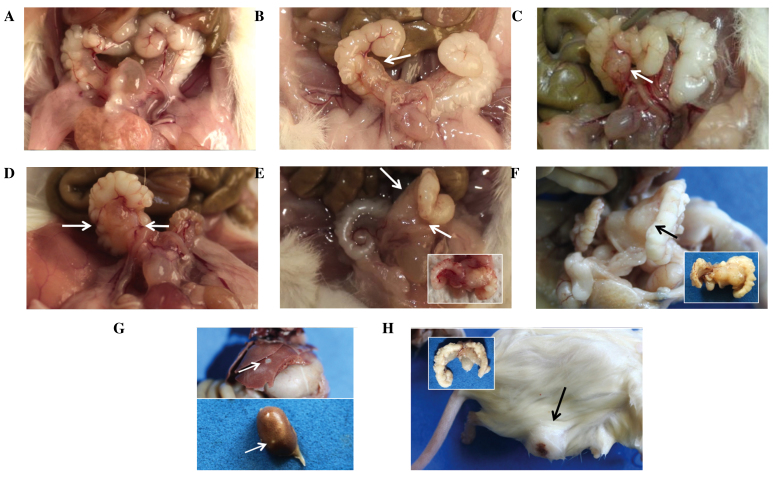
Macroscopic tumors formed as a result of the orthotopic injection of PC3 cells in the anterior prostate of NOD-SCIDγ mice. (A) A mouse from group 1 (phosphate-buffered saline) presents no visible tumors. (B) A small tumor is visible in the right anterior prostate (arrow) in a mouse from group 2 (10 days p.i.). (C) A well-delimitated tumor (arrow) is present in the mid portion of the gland in a mouse from group 3 (20 days p.i.). (D) In a mouse from group 4 (30 days p.i.), the tumor margins are poorly defined (arrows). (E) In a mouse from group 5 (45 days p.i.), the tumor (between arrows) invades the adjacent seminal vesicle. For clarity, this tumor, in the extracted anatomic piece, has been highlighted in the box. (F) The tumor in a mouse from group 6 (55 days p.i.; formalin-fixed tissue), presents similar characteristics than that in panel E, with more prominent growth and deformation of adjacent tissues (arrow). The box highlights this tumor, delineated by the dashed line. (G) Liver and kidney nodular metastases (arrows), from the mouse in panel F. (H) A mouse from group 7 (55 days p.i.; formalin-fixed tissue) presents ulceration in the skin overlying the tumor. The box exhibits the prostate of this mouse, with no evidence of tumor growth. p.i., post injection.

The animals in group 1 (PBS injection) presented no signs of tumor development. Their prostatic lobes were moderately translucent, with small blood vessels (Fig. 1A).

In the animals of groups 2–6, tumors developed at the site of injection. The tumors were white, firm and multinodular (Fig. 1B-F). The data regarding the size and extension of these tumors and their associated metastases are summarized in Table I.

Macroscopic metastases were observed in the 2 mice of group 6. Metastatic tumors (2 mm in diameter) were identified in the liver (2 tumors in one mouse and 3 tumors in the other) and kidney (1 tumor in each mouse) (Fig. 1G). These tumors were nodular, white and firm, with regular and well-defined margins.

In the mice of group 7 (subcutaneous injection), tumors developed in the site of injection. These tumors adhered to the dermis and the subcutaneous tissue, but not to the underlying muscle (Fig. 1H). The tumors were multinodular, firm and white, with yellow areas and black spots on the cut surface. In one of the mice, the epidermis was ulcerated (Fig. 1H).

#### Histology and immunohistochemistry

No changes were observed in the tissues from the mice of group 1. The immunohistochemical analysis for human mitochondria was negative in all the tissues tested from these mice.

**Figure 2.**
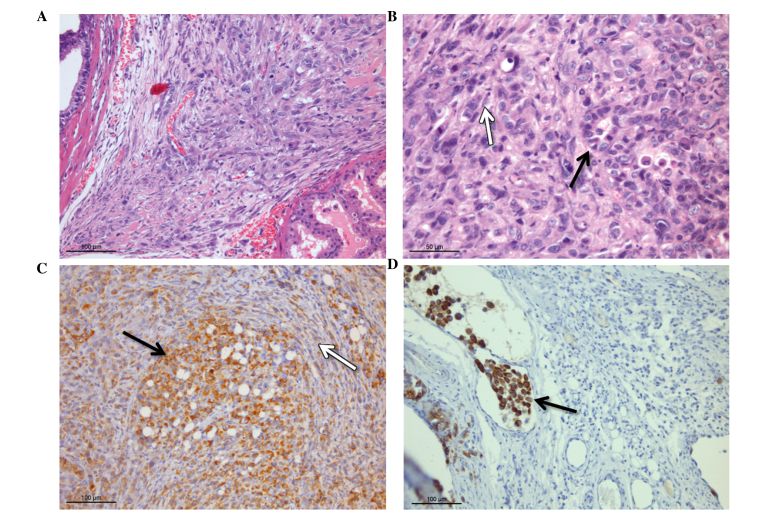
Histological and immunohistochemical analyses of the tumors that developed in the anterior prostate of NOD-SCIDγ mice following orthotopic injection of PC3 cells. (A) Mouse from group 2 (10 days p.i.). The tumor cells are located in the interstitium, compressing the adjacent tissue. (B) Mouse from group 5 (45 days p.i.). Different patterns of growth were observed, including nests and pseudoglandular configuration (black arrow), and a fusiform pattern (white arrow). (C and D) Mouse of panel B. The immunohistochemical analysis for human mitochondria, peroxidase reaction and 3,3′-diaminobenzidine chromogen indicates that the majority of the cells were positive for these markers. (C) Negative cells were considered to be part of a desmoplastic response from the mouse tissue. (D) At the periphery of the tumor, foci of vascular invasion by groups of tumor cells positive for the marker are observed. p.i., post injection.

Tumors developed in the anterior prostate of the mice of groups 2–6. The tumors had well-defined, unencapsulated margins, with invasion foci into the surrounding stroma (Fig. 2A and B). In the largest tumors (groups 4–6), malignant cells invaded non-neoplastic prostatic glands, isolating and compressing them. Foci of lymphatic invasion were observed. The tumor cells developed into 2 distinct morphological patterns, which were most evident in the largest tumors (groups 4–6) (Fig. 2B). The first pattern was most prevalent in the center of the tumors, and was characterized by the formation of nests (well-defined circular-to-polygonal groups of cells, not surrounded by connective tissue). The cells in these nests were polygonal-to-rounded, with well-defined limits and occasionally vacuolated cytoplasm. Their nuclei exhibited coarsely granular chromatin and a prominent nucleolus. In addition, marked anisokaryosis was observed. In the more advanced tumors (groups 5 and 6), a number of these nests presented clear spaces, resembling an acinar configuration. The second morphological pattern was most commonly present in the periphery of the tumors, and was occasionally continuous with invasion foci. This pattern was composed of fusiform cells organized in short, parallel bundles. The tumor cells had indistinct margins, limited cytoplasm and oval-to-elongated nuclei with coarsely granular chromatin. The mitotic index in the 2 populations of cells was similar, with 3–4 mitotic figures/high power field (magnification, ×400; field diameter, 0.53 mm; total area in 1 field, 0.22 mm^2^), including atypical mitotic figures characterized by asymmetric or non-bipolar metaphases.

The immunohistochemical analysis for human mitochondria demonstrated the human origin of the tumors (Fig. 2C), despite the fact that certain cells were negative to the marker in the prostatic tumors. A number of these cells may be mouse fibroblasts or stromal cells that are part of a desmoplastic response to the presence of tumor cells. The immunohistochemical analysis also confirmed a human origin for the cells observed inside the lymphatic vessels at the periphery of the tumor (Fig. 2D).

**Figure 3.**
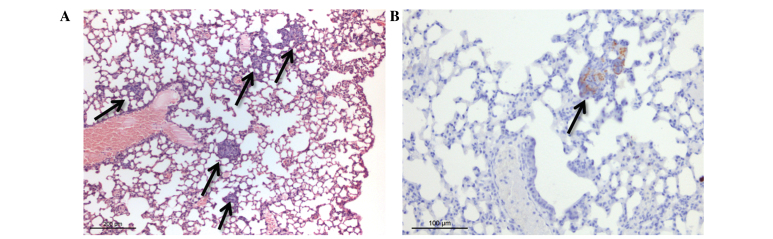
Pulmonary metastases in mice following the orthotopic injection of PC3 cells in the anterior prostate. (A) Several clusters (arrows) of tumor cells are visible surrounding an airway in a mouse from group 5 (45 days post injection). (B) The cells in the clusters were positive for the human mitochondrial marker (arrow).

In the lungs of the mice of groups 4 and 5, small aggregates of tumor cells were observed, located near the end of terminal bronchioles and under the visceral pleura (Fig. 3A). In the mice of groups 4 and 5, these aggregates were composed of 3–4 and 5–25 cells, respectively. These cells were positive for the human mitochondrial marker, confirming that they were originated from the PC3 cells (Fig. 3B).

In the animals of group 6, similar aggregates of tumor cells were observed in the lung and pancreas. No macroscopic tumors had developed in these organs by the end of the experiment.

**Figure 4.**
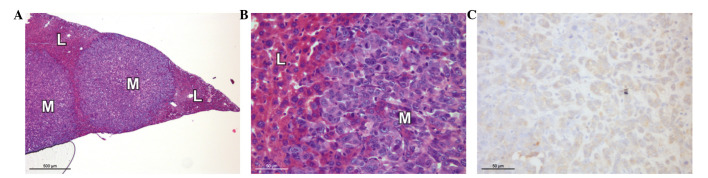
Liver metastases in mice following the orthotopic injection of PC3 cells in the anterior prostate. (A) Mouse from group 5 (45 days post injection), 2 metastatic nodules (M) are observed, compressing the neighboring hepatic parenchyma (H). (B) Detail from panel A, indicating that the tumor cells are invading several foci. (C) Immunohistochemical analysis for human mitochondria, peroxidase reaction and 3,3′-diaminobenzidine chromogen performed in the mouse from panel B demonstrates that the cells from the metastatic nodules are consistently positive for the marker.

**Figure 5.**
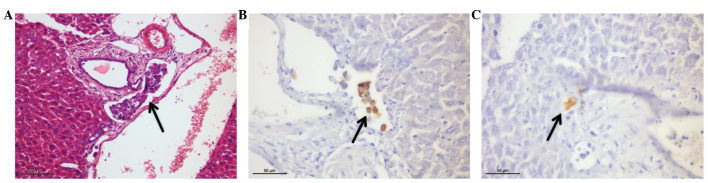
Intravascular tumor cells in mice following the orthotopic injection of PC3 cells in the anterior prostate. (A) Mouse from group 5 (45 days post injection). A tumoral embolus is observed inside an hepatic blood vessel. (B) Immunohistochemical analysis for human mitochondria, peroxidase reaction and 3,3′-diaminobenzidine chromogen performed on the tumor of panel A indicates that the tumor cells are positive for the marker. (C) The marker also identifies individual tumor cells inside the vessels.

The hepatic tumors were formed by cells (generally rounded-to-polygonal) arranged in a solid pattern (Fig. 4A and B). Renal tumors exhibited a similar morphology. The 2 patterns described for the primary tumors were present in a number of the metastases, but in a less prominent manner. The margins of the metastatic tumors were well-defined, unencapsulated and compressed the organ parenchyma, with a number of small foci of invasion (Fig. 4B). The immunohistochemical analysis for the human mitochondrial marker was positive in the majority of the cells that formed the metastatic tumors (Fig. 4C). Also, tumor cells (individual and small groups) positive for the mitochondrial marker were identified inside the blood vessels of the liver. These cells were considered to be disseminated cells from the primary tumor, suggesting that dissemination may involve groups of cells (Fig. 5A and B) or individual cells (Fig. 5C).

## Discussion

Orthotopic injection of tumor cells has previously been performed in several *in vivo* models of cancer with the aim of studying the dynamics of progression and dissemination of tumors (17,18). In PCa, orthotopic injection is usually performed in the dorsolateral prostate (7,11). To the best of our knowledge, no study using the anterior prostate for injection of PCa cells has been published thus far. This portion of the gland has a remarkable molecular similarity with the dorsolateral lobe (14), and has the advantage of allowing easy extraction of the primary tumor in order to study local relapse.

In the present study, the tumors that developed in the subcutaneous tissue (group 7) were localized, well-delimitated and non-encapsulated (Fig. 1H). These tumors presented neovascularization and foci of vascular invasion, although no macroscopic or microscopic metastases were detected. The presence of intravasation may suggest dissemination, but in the time frame of the study (55 days) this dissemination was not translated into the formation of distant tumors. By contrast, the mouse prostatic tissue may be closer to the biochemical and tissue environment of the human prostate than the subcutaneous tissue, and may allow further development of the tumors, including the ability to disseminate and metastasize successfully (13).

In the mice in groups 2–4, the prostate tumors grew slowly during the first 30 days post-injection, retaining a well-delimitated and non-encapsulated nodular morphology. This is similar to the growth rate observed in the subcutaneous tumors (Table I and Fig. 1). This growth is eminently expansive, compressing neighboring glandular structures. A small number of foci of invasion were observed. In the animals sacrificed at 45 and 55 days post injection, tumors presented a more invasive growth, surrounding and trapping a number of prostate glands. These glands were dilated, suggesting compression of glandular ducts. Also, the tumors in the animals of these groups were larger than those in groups 2–4, possibly representing a faster growth rate at more advanced stages. This situation is a common feature of spontaneous human epithelial tumors, and is one of the classical features of tumor growth (19,20).

The cell morphology was similar in all the tumors, and 2 basic morphological patterns were identified (Fig. 2B). In the first pattern, the cells were arranged in nests that occasionally presented a pseudoglandular appearance, due to the presence of small angular cavities (cribriform pattern). In human PCa, this pattern may be observed in certain metastases, simulating the epithelial pattern observed in the primary tumor (21). Also, in specific circumstances, the morphology of the metastases may resemble that of the tissue of origin, but not necessarily the morphology of the primary tumor (22). These results indicate that PC3 cells may recreate to a certain extent the morphological patterns characteristic of prostate tissue. Regardless, further studies are required to complement this hypothesis, including molecular studies aimed at identifying markers of epithelial origin.

The second pattern consisted of fusiform cells arranged in poorly-defined bundles (Fig. 2B). In the tumors developed ≤30 days post-injection (groups 2–4), the cells were mainly located in the periphery, infiltrating neighboring tissue. This pattern is morphologically similar to that of cells derived from the mesenchymal tissue.

The 2 patterns (nests and bundles) combined are morphologically similar to the highest grade in the Gleason score used in human PCa (grade 5), which represents an anaplastic, poorly differentiated pattern, usually associated with local invasion and metastasis (23).

Metastases were initially observed in the lung, at 30 days post-injection (group 4), and were consistently identified in all the animals from groups 5 and 6. These tumors were microscopic, and consisted of clusters of 5 and ≤25 cells in groups 4 and 6 (55 days post injection), respectively. The lung tumors were smaller than the metastases observed in the liver, pancreas and kidney of the mice in these groups. The lung is a frequent target for metastases, due to biomechanical factors, since it is the first capillary bed encountered by the disseminated cells that have passed through the right ventricle (24,25). This is particularly clear for metastases of lymphatic origin (25). The absence of macroscopic metastases in the lung suggests that, in this model, the lung may work as a filter organ but may lack the biological or molecular environment required to promote further tumor growth. Nonetheless, it is possible that the observed tumor cells are in a state of growth arrest (dormancy) (26), and that the time frame considered for the present study (55 days) was not sufficient for the cells to escape this blockage. Alternatively, it is also possible that the liver and kidney present a more favorable microenvironment than that offered by the lung for the disseminated cells to establish and grow.

The 2 histological patterns described for the primary tumors were present in certain liver and renal metastases, but in a less prominent manner. Differences in the cytological morphology between primary tumor cells, circulating tumor cells and disseminated tumor cells in a similar model have been reported previously, but the study involved surgical orthotopic implantation of fragments of tumors, which may account for the variability observed (27). In the present study, no cytological assessments were performed, but the presence of different histological patterns in the metastases suggests that these are initiated by ≥1 clones with different morphologies. Alternatively, it is possible that the metastases are formed by the tumor-initiating cells, which gradually generate differentiated cell populations (28).

In conclusion, the orthotopic injection of PC3 cells in the anterior prostate of NOD-SCIDγ mice results in the development of a primary tumor that has a growth rate that varies from slow at the beginning of tumor formation, to fast at the end. The prostatic tumor precedes the development of micrometastases in the lung, liver and pancreas. The tumor cells are capable of forming macroscopic tumors (macrometastases) in the liver and kidney. These cells may be identified, either individually or in groups, using a human mitochondrial marker. The primary and metastatic tumors present similar histological features, with 2 main identifiable morphological patterns.

The modification of the orthotopic model described in the present study may represent a valuable tool for the study of cytoreductive surgery and the recurrence of PCa.

## Figures and Tables

**Table I.** Summary of animal groups, experimental conditions and results from orthotopic or subcutaneous injection of PC3 cells in NOD-SCIDγ mice.

Group no.	Injection site	End day of experiment	Final tumor size (mm)	Prostate tumor extension/invasion	Metastases
1	AP	55	No tumor	-	-
2	AP	10	2–2.5	Well delimitated nodular tumor	-
3	AP	20	5–6	Well delimitated nodular tumor	-
4	AP	30	7.5–8.6	Poorly delimitated irregular tumor; ipsilateral SV invasion; prominent blood vessels	Lung micrometastases
5	AP	45	6–6	Poorly delimitated irregular tumor; ipsilateral SV invasion; prominent blood vessels	Lung micrometastases
6	AP	55	8–8	Poorly delimitated irregular tumor; contralateral SV invasion; prominent blood vessels	Lung and pancreas micrometastases; liver and kidney macrometastases
7	SC (right flank)	55	10–13	-	-

N=2 mice/group. Mice in group 1 were administered PBS, whereas the mice in groups 2–7 were injected with 10^6^PC3 cells suspended in phosphate-buffered saline in a total volume of 50 µl. AP, anterior prostate; SC, subcutaneous; SV, seminal vesicle.
